# A Combined Nucleic Acid and Protein Analysis in Friedreich Ataxia: Implications for Diagnosis, Pathogenesis and Clinical Trial Design

**DOI:** 10.1371/journal.pone.0017627

**Published:** 2011-03-11

**Authors:** Francesco Saccà, Giorgia Puorro, Antonella Antenora, Angela Marsili, Alessandra Denaro, Raffaele Piro, Pierpaolo Sorrentino, Chiara Pane, Alessandra Tessa, Vincenzo Brescia Morra, Sergio Cocozza, Giuseppe De Michele, Filippo M. Santorelli, Alessandro Filla

**Affiliations:** 1 Department of Neurological Sciences, University Federico II, Naples, Italy; 2 Molecular Medicine, IRCCS Stella Maris, Pisa, Italy; 3 Department of Cellular and Molecular Biology, University Federico II, Naples, Italy; Brigham and Women's Hospital, Harvard Medical School, United States of America

## Abstract

**Background:**

Friedreich's ataxia (FRDA) is the most common hereditary ataxia among caucasians. The molecular defect in FRDA is the trinucleotide GAA expansion in the first intron of the *FXN* gene, which encodes frataxin. No studies have yet reported frataxin protein and mRNA levels in a large cohort of FRDA patients, carriers and controls.

**Methodology/Principal Findings:**

We enrolled 24 patients with classic FRDA phenotype (cFA), 6 late onset FRDA (LOFA), all homozygous for GAA expansion, 5 pFA cases who harbored the GAA expansion in compound heterozygosis with *FXN* point mutations (namely, p.I154F, c.482+3delA, p.R165P), 33 healthy expansion carriers, and 29 healthy controls. DNA was genotyped for GAA expansion, mRNA/*FXN* was quantified in real-time, and frataxin protein was measured using lateral-flow immunoassay in peripheral blood mononuclear cells (PBMCs). Mean residual levels of frataxin, compared to controls, were 35.8%, 65.6%, 33%, and 68.7% in cFA, LOFA, pFA and healthy carriers, respectively. Comparison of both cFA and pFA with controls resulted in 100% sensitivity and specificity, but there was overlap between LOFA, carriers and controls. Frataxin levels correlated inversely with GAA1 and GAA2 expansions, and directly with age at onset. Messenger RNA expression was reduced to 19.4% in cFA, 50.4% in LOFA, 52.7% in pFA, 53.0% in carriers, as compared to controls (p<0.0001). mRNA levels proved to be diagnostic when comparing cFA with controls resulting in 100% sensitivity and specificity. In cFA and LOFA patients mRNA levels correlated directly with protein levels and age at onset, and inversely with GAA1 and GAA2.

**Conclusion/Significance:**

We report the first explorative study on combined frataxin and mRNA levels in PBMCs from a cohort of FRDA patients, carriers and healthy individuals. Lateral-flow immunoassay differentiated cFA and pFA patients from controls, whereas determination of mRNA in q-PCR was sensitive and specific only in cFA.

## Introduction

Friedreich's ataxia (FRDA), an autosomal recessive neurodegenerative disorder, is the most common hereditary ataxia among Caucasians [1]. The disease is characterized by gait and limb ataxia, dysarthria, usually absent tendon reflexes, bilateral Babinski sign, impairment of position and vibratory senses, scoliosis, and pes cavus [2]. Cardiomyopathy is the predominant cause of death [3].

The molecular defect in FRDA is the trinucleotide GAA expansion in the first intron of the *FXN* gene [4]. Most patients are homozygous for this mutation. Two to 5% of patients harbor a point mutation on one allele and a GAA expansion on the other allele. The *FXN* gene encodes a 210 amino acid mitochondrial protein named frataxin. *FXN* mRNA was found to be reduced to 13–30% in FRDA patients, and to 40% in carriers, as compared to control mRNA [5]. The residual amount of frataxin protein in FRDA patients varies between 4 and 29% of the level seen in normal control, and shows an inverse correlation with the size of the GAA1 repeat [6]. Although the exact physiological function of frataxin is not known, its involvement in iron–sulphur (Fe–S) cluster and heme biogenesis, iron binding/storage and iron chaperone activity has been suggested [7], [8], [9].

To date four studies have precisely quantified frataxin levels in FRDA patients, carriers or controls. A first study [10] adopted a lateral flow immunoassay to quantify frataxin in peripheral blood mononuclear cells (PBMC), cultured lymphoblasts, and cheek swabs. Standard curves were prepared using recombinant frataxin (amino acids 56-210). In that study, frataxin was also determined in lymphoblastoid cell lines from five controls, four carriers, and seven FRDA patients. The control mean±SD frataxin level was 438±62 pg frataxin per µg total cell protein (range 343–488). Residual protein levels were 64% in carriers, and 29% in FRDA patients and there was overlap between FRDA patients and carriers. A second study determined frataxin in cheek swabs by lateral flow immunoassay [11]. FRDA patients showed 20.9%, and carriers 50.2% of frataxin levels of controls. Similar data were obtained in whole blood samples. A recent quantitative electrochemiluminiscence assay (ECLIA) [12] measured 7.9–11.9 in PBMC from five controls and 1.1–4.8 pg/µg frataxin in 11 FRDA patients (reduction to 27% of controls), and showed no overlap between the two groups.

Even more recently, the same group showed that frataxin levels ranged 0.056–0.169 pg/µg protein when assayed using an in-house enzyme-linked immunosorbent assay (ELISA) [13]. There was no correlation between frataxin levels, size of GAA repeats, age, and gender in that study.

The aim of the present study was to combine the determination of frataxin levels and mRNA expression in PBMCs from a cohort of consecutive FRDA patient, carriers, and controls, and to correlate results with genotype and clinical presentation.

## Materials and Methods

### Study Design

We designed an observational study to examine frataxin levels in FRDA patients, FRDA carriers, and controls. The local Ethics Committee of our Institution, “Comitato Etico per le Attività Biomediche dell'Università degli Studi di Napoli Federico II”, approved the clinical trial (registration number 49/09). All patients gave written informed consent before any activity linked to the study was started. Patients, carriers, and controls were consecutively enrolled from March 2009 to April 2010. Patients were divided in the following categories. Classic FRDA (cFA) was defined as patients with a molecular diagnosis of FRDA with a number of GAA triplets within the pathological range on both alleles [4], and an onset before the age of 25 years. Point mutation FRDA (pFA) was defined as patients with a number of GAA triplets within the pathological range on one allele, and a point mutation on the other allele. Late onset Friedreich Ataxia (LOFA) was defined as patients with age at onset ≥25 years [14], [15] and a molecular diagnosis of FRDA with a number of GAA triplets within the pathological range on both alleles. Carriers were selected on an obligate carrier basis. Controls were randomly enrolled through the site personnel of our University.

### Quantitative analysis of Frataxin with Lateral Flow Immunoassay

PBMCs were extracted from 30 mL of EDTA anticoagulated whole blood using Leucosep® tubes (Greiner bio-one, Frickenhausen, Germany) and frozen at −80°C until analysis. PBMCs were lysed and total protein was measured using the bicinchoninic acid assay. In each well, 7.125 µg of total protein extract were loaded. Dipsticks were added and processed following kit instructions. Dried dipsticks were analyzed with the Hamamatsu ICA-1000 scanner and quantified with dedicated software. Interpolation was performed on a standard curve constructed with recombinant human frataxin (extraction kit, dipsticks and scanner, Mitosciences, Eugene, OR, USA) [10]. In our hands, lateral flow immunoassay performed well, with intra-assay and inter-assay coefficients of variability both <5%.

### Molecular analysis of GAA expansion

DNA used for PCR amplification was extracted from venous blood leukocytes using standard methods. Amplification of normal and expanded alleles was obtained by PCR procedures previously described [16]. Triplet Repeat Primed PCR (TP-PCR) was performed according to the protocol previously described [17]. Briefly, primer sequences used for FRDA TP PCR test were: P1 5′-GCTGGGATTACAGGCGCGCGA-3′, P3 5′- TACGCATCCCAGTTTGAGACG-3′, P4 5′-6-FAM TACGCATC-CCAGTTTGAGACGGAAGAAGAAGAAGAAGAAGAA-3′. TP-PCR assay was performed in a reaction volume of 15 µl containing 100 ng genomic DNA, 1.5 mmol/L MgCl_2_, 10 mmol/L Tris (pH 8.3), 50 mmol/L KCl, 0.8 µmol/L primer P1, 0.7 µmol/L primer P3, 0.07 µmol/L primer P4, 200 µmol/L dNTPs each, and 2 units *Taq* polymerase. The reactions were subjected to 30 cycles consisting of 94°C for 30 seconds, 60°C for 30 sec, 72°C for 30 sec followed by a 10 min extension at 72°C. PCR products were incubated at 95°C for 2 min and cooled on ice before loading and resolved by electrophoresis on an automatic sequencer (ABI 3500; Applied Biosystems, Foster City, CA). Two µl of each PCR product were added to 18 µl of formamide (Applied Biosystems, Foster City, CA) and 0.5 µl of Genescan 400HD (Rox) Size standard (Applied Biosystems). Each sample underwent TP- PCR three times. The size of the PCR product was estimated using appropriate DNA molecular marker size standards (Invitrogen, San Diego, CA, USA) on agarose 0.8% gel stained with ethidium bromide using ImageQuant TL software in an ImageQuant 350 molecular imager (GE Healthcare Europe GmbH, Freiburg, Germany).

Direct gene sequencing of *FXN* used primers and PCR conditions reported elsewhere [4].

### Quantitative real time PCR (q-PCR) of frataxin mRNA

Total mRNA was extracted from PBMCs using TRIzol® reagent (Invitrogen) following manufacturer's instructions. RNA was spectrophotometrically quantified, and qualitative analysis was performed with agarose-formaldehyde electrophoresis and ethidium bromide staining. One µg mRNA was reversely transcribed using the one-step High Capacity RNA-to-cDNA Master Mix (Ambion, Applied Biosystems, Carlsbad, CA, USA) following manufacturer's instructions in a total volume of 20 µL. About 2 µL of cDNA were amplified using the TaqMan® Gene Expression Master Mix and TaqMan® Gene Expression Assay for frataxin (Applied biosystems, catalog n°Hs00175940_m1) in a StepOne real-time PCR. RNA was standardized by quantification of hypoxanthine phosphoribosyl-transferase 1 (HPRT1) as a reference gene (Applied Biosystems, TaqMan® Endogenous controls). Relative expression was calculated with the efficiency calibrated model [18].

### Statistical Analysis

Statistical analysis for continuous variables was conducted by one-way ANOVA. Post-hoc analysis was performed with the Bonferroni multiple comparison's test. Diagnostic efficacy of frataxin dosage was assessed constructing ROC curves and calculating area, sensitivity, specificity and P value. Correlation analysis was performed calculating Pearson's coefficient. Normality tests were performed with the Kolmogorov-Smirnov test. P values of less that 0.05 were considered statistically significant. Analysis was performed using the Prism software version 5.0c for MAC (Graphpad, La Jolla CA, USA). Quantitative-PCR data in real-time were analyzed with the improved version of the relative expression software tool (REST 2009) [18].

## Results

### Patients

A total of 38 patients were enrolled in this study. Twenty-seven patients were cFA, 5 pFA, and 6 LOFA (Table 1). Patients with point mutations (Table 2) have been previously described [19], [20]. Thirty-three FRDA carriers and 30 healthy controls were also enrolled in the study. GAA triplets were measured in all patients, carriers and controls. One obligate carrier was found to have normal GAA repeats on both alleles, and one control showed a GAA expansion on one allele. According to their unexpected genotype, both individuals were shifted to the correct group and further analyzed.

**Table 1:** Demographics and genotyping for all subjects.

	Number	Age (years)	Onset(years)	DiseaseDuration(years)	GAA1 repeats	GAA2 repeats
cFA	27	35±12	15±8	20±10	755±292	948±254
pFA	5	42.0±5	9.0±4	33±3	905±101	-
LOFA	6	49±8	35.0±10	14.0±7	375±127	753±262
Carriers	33	51±17	-	-	15±7	983±440
Controls	30	32±9	-	-	11±6	12±6

Classic FRDA (cFA), point mutation FA (pFA), late onset FA (LOFA), carriers, and controls. All data are presented as mean±SD.

**Table 2:** Point mutation FA patients.

Patient	Sex	Mutation	GAA repeats	Age	Age at onset	Disease duration	Frataxin protein pg/µg total protein	Frataxin protein %	Frataxin mRNA %
1	M	p.I154F	959	42	6	36	3.60	9.3	33
2	M	p.I154F	921	44	13	31	5.20	13.5	48
3	M	c.482+3delA	731	46	9	37	10.34	26.8	52
4	F	p.R165P	988	34	4	30	21.10	54.7	67
5	F	p.R165P	926	43	10	33	23.40	60.6	75

Demographics, neurological data, frataxin protein and *FXN* mRNA levels in pFA. Frataxin protein % and *FXN* mRNA levels % are expressed as relative to the control group.

### Frataxin levels

**Figure 1:**
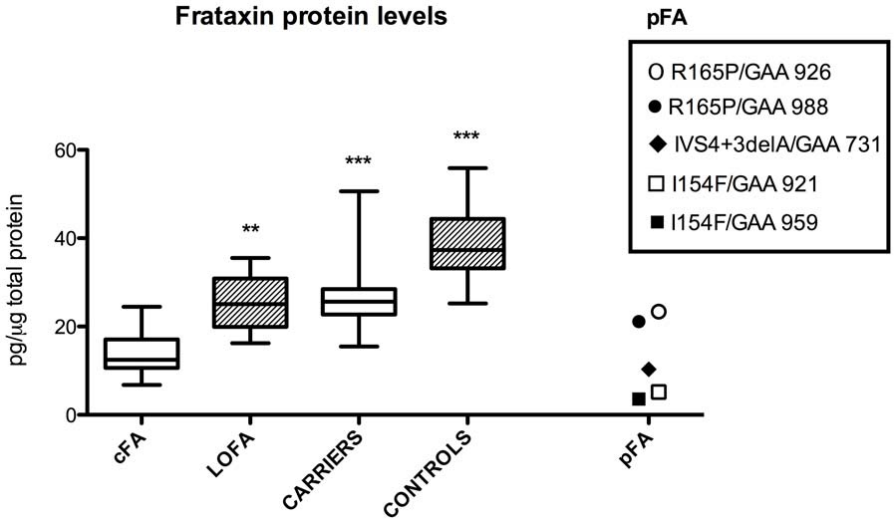
Frataxin protein levels in PMBCs. Box and wiskers plot (min to max) of frataxin levels in cFA (n = 24), LOFA (n = 5), carriers (n = 33), controls (n = 29), and pFA (n = 5). Statistical significance is indicated after comparison to cFA (**p>0.01, ***p<0.001).

Mean±SD frataxin in cFA patients was 13.8±4.6 pg/µg total protein, and ranged from a minimum of 6.8 to a maximum of 24.5 (Figure 1). The five point mutation patients had frataxin levels of 12.7±9.1 pg/µg total protein (range 3.6–23.4; Table 2). LOFA patients showed mean level of 25.3±6.9 (range 16.2–35.5) whereas frataxin level was 26.5±7.1 (15.5–50.6) in carriers and 38.6±7.6 (25.3–55.9) in healthy controls. Mean residual levels of frataxin, as compared to control level, were 35.8, 33.0, 65.6, and 68.7% for cFA, pFA, LOFA, and carriers, respectively.

Comparison of the different groups showed a significant difference between cFA patients compared with LOFA, carriers and controls (Figure 1; p<0.01, p<0.001, p<0.001). Similar results were obtained comparing pFA patients with LOFA, carriers, and controls.

### Diagnostic efficiency of frataxin measurement

ROC curves were constructed to assess the efficacy of frataxin dosage to discriminate between groups (Figure S1). Comparison of cFA patients vs controls, resulted in a 100% sensitivity and specificity for a cut-off frataxin value of 24.8 pg/µg total protein (p<0.0001, area = 1.00). Similar results were obtained comparing pFA patients with controls with the same frataxin cut-off value (p<0.001, area = 1.00). Comparison of cFA patients, vs carriers resulted in a sensitivity of 100% and specificity of 70.83% for a cut-off value of 15.27, and a sensitivity of 66.67% and specificity of 100% for a cut-off value of 24.54 (p<0.0001, area = 0.9596). Comparison of carriers vs controls resulted in a useless sensitivity of 100% and specificity of 45.45% for a cut-off value of 25.19 (area = 0.8945).

### Frataxin Correlations

**Figure 2:**
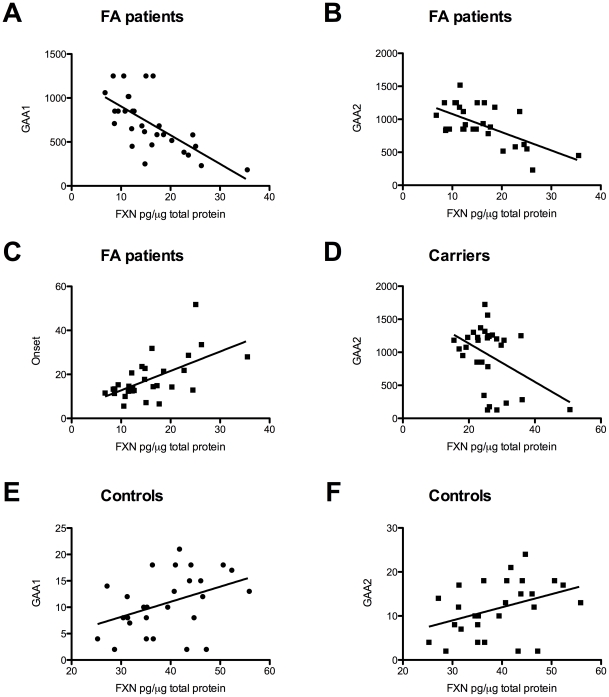
Protein correlation analysis. A) correlation between frataxin levels and GAA1 repeats in cFA and LOFA pateints (p<0.0001, R^2^ = 0.4632); B) correlation between frataxin levels and GAA2 repeats in cFA and LOFA patients (p<0.001, R^2^ = 0.3886); C) correlation between frataxin levels and age at onset for cFA and LOFA (p<0.001, R^2^ = 0.3577); D) correlation between frataxin and GAA2 in carriers (p<0.05, R^2^ = 0.1745); E) correlation between frataxin levels and GAA1 in controls (p<0.05, R^2^ = 0.1634); F) correlation between frataxin levels and GAA2 in controls (p<0.05, R^2^ = 0.1463).

For correlation analysis cFA and LOFA were considered together (simply termed as FRDA patients). In FRDA patients, GAA1 and GAA2 correlated inversely with frataxin levels (p<0.0001, R^2^ = 0.4632; P<0.001, R^2^ = 0.3886; Figure 2A–B), whereas age at onset correlated directly with frataxin levels (p<0.001, R^2^ = 0.3577; Figure 2C). In obligate carriers, we found a mild inverse correlation between the size of the mutated (p<0.05, R^2^ = 0.1745; Figure 2D) but not of the wild-type (p = 0.7, R^2^ = 0.006) allele and frataxin levels. Unexpectedly, control levels of frataxin correlated directly with both the GAA1 and GAA2 (p<0.05, R^2^ = 0.1634; p<0.05, R^2^ = 0.1463; Figure 2E–F).

### q-PCR of FXN mRNA

**Figure 3:**
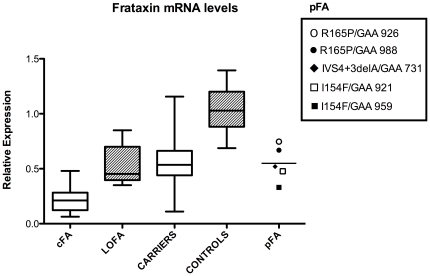
*FXN* mRNA levels in PBMC. Box and wiskers plots (min to max) of *FXN* mRNA relative expression levels in cFA (n = 23), LOFA (n = 6), carriers (n = 29), controls (n = 25), and pFA (n = 5). Statistical significance for all groups compared to controls is p<0.0001.

In cFA, mRNA was profoundly reduced to 19.4% of controls (range 0.06–0.48, p<0.0001, Figure 3,) whereas there was a less severe down-regulation in pFA (52.7% of controls, p<0.0001, Table 2). This resulted in different mRNA relative expression levels between cFA and pFA (p<0.001). LOFA patients and carriers showed a less severe down-regulation to 50.4% (range 0.35–0.85, p<0.0001) and 53.0% (range 0.11–1.21, p<0.0001), respectively. Difference was also significant when comparing mRNA levels between cFA and LOFA (p<0.0001), or carriers (p<0.0001). In contrast, there was no difference between LOFA or pFA and carriers (p = 0.914 and p = 0.890, respectively).

### Diagnostic efficiency of FXN mRNA expression

ROC curves were constructed, similarly to frataxin protein levels, to assess the efficacy of *FXN* mRNA relative expression to discriminate between groups. Comparison of cFA patients vs controls, resulted in a 100% sensitivity and specificity for a cut-off mRNA value of 0.6 as compared to controls (Figure S1; p<0.0001, area = 1.00). Comparison of pFA with controls resulted in a sensitivity of 100% and specificity of 88% for a cut-off frataxin mRNA value of 0.75 (p<0.001, area = 0.976). Comparing cFA patients vs. carriers, and carriers vs controls gave similar results as for frataxin protein and did not reach a useful sensitivity and specificity.

### Frataxin mRNA Correlations

**Figure 4:**
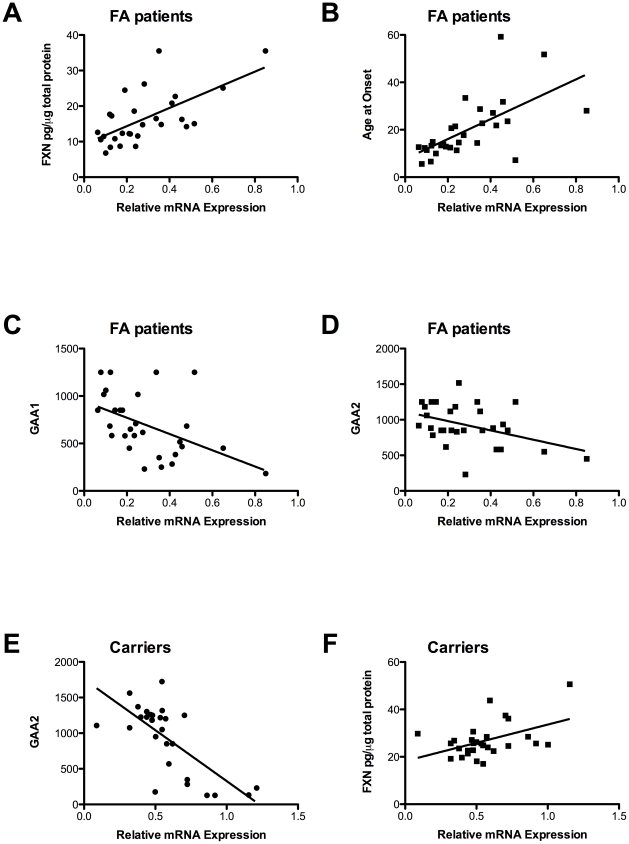
*FXN* mRNA correlation analysis. A) correlation between *FXN* mRNA and protein levels cFA and LOFA patients (p<0.001, R^2^ = 0.4058); B) correlation between *FXN* mRNA levels and age at onset in cFA and LOFA patients (p<0.0001, R^2^ = 0.3905); C) correlation between *FXN* levels and GAA1 for cFA and LOFA (p<0.01, R^2^ = 0.2364); D) correlation between *FXN* levels and GAA2 for cFA and LOFA (p<0.05, R^2^ = 0.1750); E) correlation between *FXN* mRNA levels and GAA2 for carriers (p<0.0001, R^2^ = 0.4667); F) correlation between *FXN* mRNA and protein levels in carriers (p<0.02, R^2^ = 0.2167).

For mRNA correlation analysis, cFA and LOFA were considered together (as FRDA patients). Messenger RNA levels correlated directly with protein levels and age at onset (p<0.001, R^2^ = 0.4058; P<0.001, R^2^ = 0.3905; Figure 4A–B) and inversely with GAA1 and GAA2 sizing (p<0.01, R^2^ = 0.2364; p<0.05, R^2^ = 0.1750, Figure 4C–D). We found an inverse correlation between mRNA levels and GAA2 in carriers (p<0.0001, R^2^ = 0.5011; Figure 4E). Mild direct correlation was found between mRNA and protein in carriers (p<0.02, R^2^ = 0.2117; Figure 4F).

### Non Gaussian distributions for carriers

**Figure 5:**
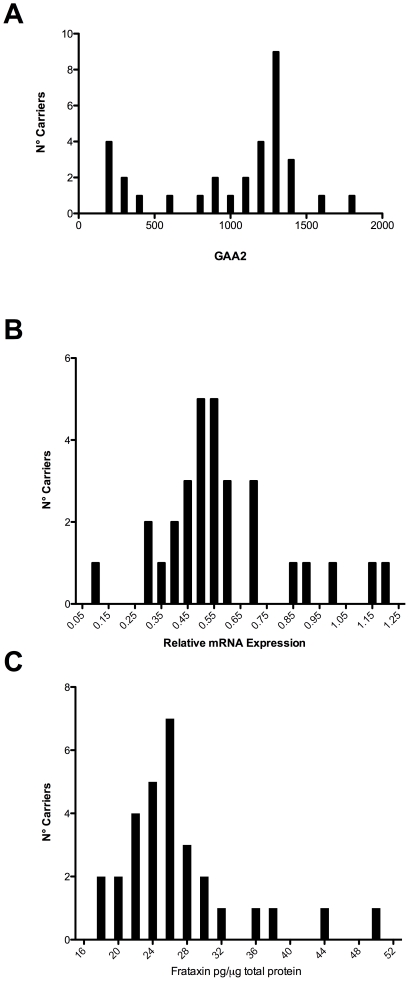
Non normal distribution of GAA2, *FXN* mRNA and frataxin protein in carriers. A) Bimodal distribution of GAA2 repeats in carriers; B) skewed distribution of *FXN* mRNA levels in carriers; C) skewed distribution of frataxin protein levels in carriers.

GAA distribution was Gaussian for all groups except for carriers, where GAA2 was bimodal (Fig 5A). This was confirmed using the Kolmogorov-Smirnov normality test (p<0.001), which showed a non Gaussian distribution of GAA2. A total of seven carriers showed <500 GAA2 repeats influencing distribution. Of these, four were related to LOFA patients, two were related to cFA, and one was initially included as a control and later found to be a carrier. A non Gaussian distribution was found for *FXN* mRNA levels (p<0.02; Fig 5B) and frataxin protein in carriers (p<0.01; Fig 5C), without a clear bimodal distribution.

## Discussion

We report the first combined study on frataxin protein and mRNA levels in PBMCs from a large cohort of FRDA patients, carriers and healthy individuals. We confirmed previous findings of reduced levels of frataxin in FRDA patients. In our study residual frataxin levels were 35.8% of mean control levels in cFA, and 68.7% in carriers. These levels are slightly dissimilar to those previously reported in FRDA patients (range 21–29%) [10]–[12], and carriers (range 50.2–64%) [10], [11].

In contrast absolute levels of frataxin showed considerable differences between laboratories. In our study, FRDA patients had average frataxin levels of 13.8±4.6 pg/µg total protein, which were different to those detected by others in PBMCs (1.1–4.8 pg/µg) [12] or lymphoblasts (127±77 pg/µg) [10], or even in control buccal swabs (30 pg/µg) [11]. This difference might be related to different recombinant frataxin used to construct the standard curve in the first study, or in the different cell type used in the second study (i.e. PBMCs vs lymphoblasts, buccal swabs, or whole blood). Thus, it seems more cautious to show data both as absolute and relative values compared to a healthy control population for each laboratory.

In this study, we also showed that mRNA levels were reduced to 19.4% in cFA, 50.4% in LOFA and 53% in carriers. Previous data had reported down-regulation in cFA (16.6%), LOFA (21.5%), and carriers (35.2%) [5]. This may be because of a different cut-off when considering the age at onset of LOFA (≥25 years in the present study and ≥20 in [5]), and because of the different samples size (5 cFA, 5 LOFA, 3 carriers and 3 controls in [5]).

Our study suggests that the absolute value of the frataxin protein and mRNA are not the only determinant of the disease. There was a strong overlap between groups in both protein and mRNA expression levels. Indeed, some healthy carriers shared the same levels with FRDA patients for both protein and mRNA, and even more intriguingly there was a clear overlap between LOFA and controls. It is tempting to speculate that low frataxin protein and mRNA levels are “condicio sine qua non” but not the only determinant for disease manifestations. It would be interesting to test individuals with different genotypes (i.e. patients and carriers, or LOFA and controls) but overlapping frataxin or mRNA levels, and if this correlates with different cellular phenotypes.

A recent paper reported that the FXN46-210 isoform is preferentially reduced in FRDA patients, and that the molar ratio between the FXN46-210 and the FXN81-210 form is altered [21]. Therefore, specific measurement of these isoforms in large cohorts of FRDA patients could be used to increase the discriminating ability of frataxin protein measurement. Nevertheless, difference in isoform levels should be confirmed in future studies, and it should be further investigated weather the FXN46-210 isoform is a simple precursor or plays a clear physiological role. In addition, data from our study should be considered prudently since levels in PBMCs may not be the same as in affected cells.

We report frataxin protein levels in five patients carrying three different point mutations. The group of pFA showed a lower age at onset when compared to cFA, despite similar frataxin protein levels, and higher mRNA levels. Frataxin protein levels were similar in siblings carrying the same point mutation and different between mutations. This was confirmed at the mRNA level. In our study pFA carrying the p.I154F showed the lowest levels of all tested patients, c.482+3delA had intermediate levels, and the two p.R165P patients had the highest levels. This is confirmed by the clinical presentation of FRDA patients carrying the p.I154F mutation, which showed earlier onset and a more aggressive phenotype when compared to p.R165P patients [19], [20]. In addition, higher GAA repeats were associated with lower frataxin protein and mRNA levels between siblings harboring the p.I154F and p.R165P mutations.

Interestingly, protein levels of all pFA were similar to cFA and could be differentiated from controls. Conversely, mRNA levels of pFA overlapped with carriers. This may be explained by the absence of a pathological effect of point mutations on frataxin transcription, while, alike carriers, the GAA expansion on the other allele reduces mRNA efficiency. It is likely that at the protein level, a given point mutation may affect frataxin stability reducing its level in PBMCs, as suggested by previous studies [22]. These results are similar to other studies that tested frataxin levels by Western blotting in lymphoblasts from patients with different point mutations (namely, p.W173G, c.157delC, c.100delC, p.Y118X, c.104delC) and showed reduced protein expression (range 6.9% and 30.9% of controls) [23].

Inverse correlation was found between frataxin protein level and GAA1 and GAA2, whereas a direct correlation was evident between frataxin and age at onset. This is in accordance with other studies [24]. Similar results were obtained with mRNA measurements.

Size of GAA2 correlated inversely with frataxin levels in carriers suggesting that, at the heterozygous state, GAA2 is able to influence residual protein even in the presence of a normal allele. In addition, GAA2 repeat number distribution was found to be bimodal in carriers. This corresponded to a similar non Gaussian frataxin mRNA and protein distribution. Although LOFA-carriers might have contributed to the distribution, a selection bias can be ruled out since there was not a preferential selection during recruitment. This is the first report on GAA2 distribution on carriers. Although preliminary and far to being predictive of the offspring's phenotype, it could be speculated that pathological GAA expansions in carriers may preferentially exist in two forms. A less frequent form (with <500 triplets) seems to be associated with LOFA, and a more frequent (>500 triplets) with cFA.

Surprisingly controls showed a significant positive correlation between GAA1 and GAA2 with frataxin levels. This finding is different from data reported by others [13] but this might be related to differences in methodological determination of protein dosage, or a higher dispersion of GAA sizes (2–21 in our study and 7–18 in [13] for GAA1; 2–24 in our sample and 8–26 in [13] for GAA2) with possible ethnic differences.

In summary, we demonstrated that frataxin protein and mRNA measurement may discriminate FRDA patients and healthy individuals, although a few limitations to the method should be taken into account. First, a LOFA phenotype cannot be predicted on the basis of protein and mRNA load, at least in PBMCs, since there was a strong overlap with carriers and controls. Second, we and others analyzed a representative though limited number of FRDA patients. Replicate studies on larger samples are needed before translation of protein or mRNA measurements into daily clinical practice. Thus, it is wiser to account on routine genetic testing for diagnosis whereas frataxin protein and mRNA may be attractive biomarkers for clinical trials [25]–[26]. Third, and counter intuitively, frataxin protein in PBMCs seem appealing in discriminating pFA with controls. Compared to the more expensive and laborious gene sequencing or Western blotting, the use of lateral-flow immunoassay in PBMCs can be proposed prior to full *FXN* sequencing if genetic testing reveals a heterozygous expansion, family history is dark, and clinical symptoms are not fully manifested.

## Supporting Information

Figure S1 ROC curves of frataxin and FXN mRNA. ROC curves showing specificity and sensitivity of frataxin measurement to discriminate between groups using frataxin protein measurement (A-D) or mRNA (E-H). A) cFA compared to controls; B) pFA compared to controls; C) cFA compared to controls; and D) carriers compared to controls; E) cFA compared to controls; F) pFA compared to controls; G) cFA compared to LOFA; and H) carriers compared to controls.
(PDF)
