# Supplementary figures and images for: A Combined Nucleic Acid and Protein Analysis in Friedreich Ataxia: Implications for Diagnosis, Pathogenesis and Clinical Trial Design
Source: PLoS One. 2011 Mar 11;6(3):e17627. doi: 10.1371/journal.pone.0017627 (PMC3055871; doi:10.1371/journal.pone.0017627)

**A**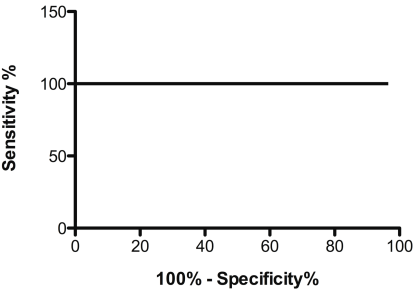**B**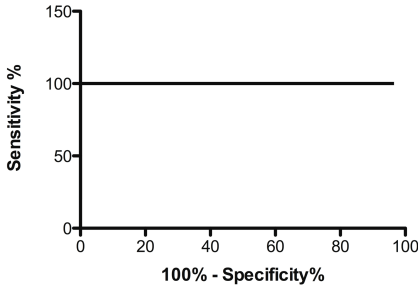**C**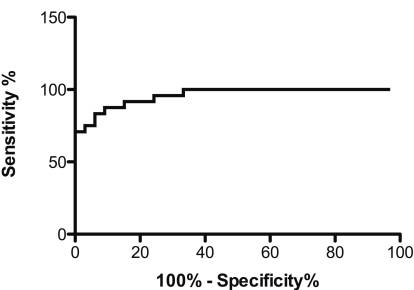**D**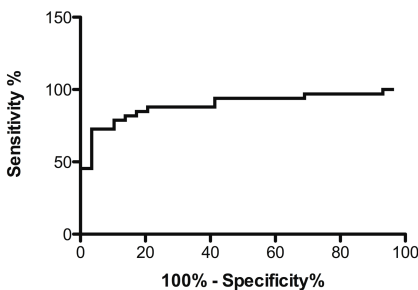**E**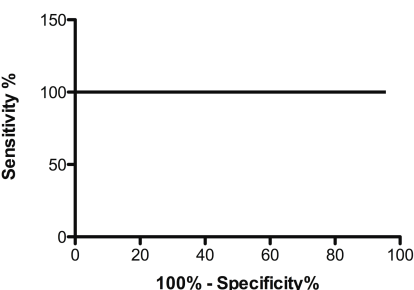**F**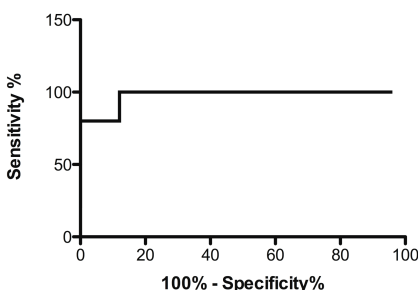**G**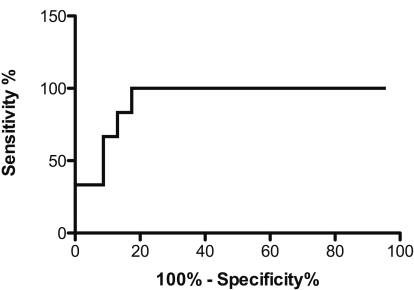**H**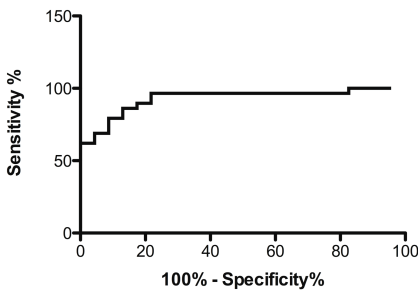

Supplement: Figure S1 — ROC curves of frataxin and FXN mRNA. ROC curves showing specificity and sensitivity of frataxin measurement to discriminate between groups using frataxin protein measurement (A-D) or mRNA (E-H). A) cFA compared to controls; B) pFA compared to controls; C) cFA compared to controls; and D) carriers compared to controls; E) cFA compared to controls; F) pFA compared to controls; G) cFA compared to LOFA; and H) carriers compared to controls. (PDF) [file pone.0017627.s001.pdf]
